# Seroprevalence of SARS-CoV-2 Assessed by Four Chemiluminescence Immunoassays and One Immunocromatography Test for SARS-Cov-2

**DOI:** 10.3389/fpubh.2021.649781

**Published:** 2021-04-29

**Authors:** Pellegrino Cerino, Alfonso Gallo, Biancamaria Pierri, Carlo Buonerba, Denise Di Concilio, Maria Concetta Cuomo, Lucia Vassallo, Gabriella Lo Conte, Annachiara Coppola, Antonio Pizzolante, Giovanni Boccia, Veronica Ferrucci, Luigi Atripaldi, Maria Triassi, Daniela Pacella, Michele Cennamo, Paolo Romano, Teresa Maria Sorbo, Alessandro Furno, Oriana Catapano, Aldo Contina, Giuseppe Perruolo, Maurizio D'Amora, Daniela Terracciano, Giuseppe Portella

**Affiliations:** ^1^Centro di Referenza Nazionale per l'Analisi e Studio di Correlazione tra Ambiente, Animale e Uomo, Istituto Zooprofilattico Sperimentale del Mezzogiorno, Portici, Italy; ^2^Department of Medicine, Surgery, and Dentistry ‘Scuola Medica Salernitana’, University of Salerno, Baronissi, Italy; ^3^Regional Reference Center for Rare Tumors, Department of Oncology and Hematology, Azienda Ospedaliera Universitaria Federico II of Naples, Naples, Italy; ^4^CEINGE Biotecnologie Avanzate, Naples, Italy; ^5^Cotugno Hospital, Azienda Ospedaliera di Rilievo Nazionale Ospedali dei Colli, Naples, Italy; ^6^Department of Public Health, Section of Hygiene, University of Naples Federico II, Naples, Italy; ^7^Department of Translational Medical Sciences, University of Naples Federico II, Naples, Italy; ^8^Unità Operativa Complessa Medicina di laboratorio P. O. San Paolo, Azienda Sanitaria Locale Naples 1 Centro, Naples, Italy

**Keywords:** SARS-CoV-2, serological test, seroprevalence, immunoassays, rapid tests

## Abstract

The onset of the new SARS-CoV-2 coronavirus encouraged the development of new serologic tests that could be additional and complementary to real-time RT-PCR-based assays. In such a context, the study of performances of available tests is urgently needed, as their use has just been initiated for seroprevalence assessment. The aim of this study was to compare four chemiluminescence immunoassays and one immunochromatography test for SARS-Cov-2 antibodies for the evaluation of the degree of diffusion of SARS-CoV-2 infection in Salerno Province (Campania Region, Italy). A total of 3,185 specimens from citizens were tested for anti-SARS-CoV-2 antibodies as part of a screening program. Four automated immunoassays (Abbott and Liaison SARS-CoV-2 CLIA IgG and Roche and Siemens SARS-CoV-2 CLIA IgM/IgG/IgA assays) and one lateral flow immunoassay (LFIA Technogenetics IgG–IgM COVID-19) were used. Seroprevalence in the entire cohort was 2.41, 2.10, 1.82, and 1.85% according to the Liaison IgG, Abbott IgG, Siemens, and Roche total Ig tests, respectively. When we explored the agreement among the rapid tests and the serologic assays, we reported good agreement for Abbott, Siemens, and Roche (Cohen's Kappa coefficient 0.69, 0.67, and 0.67, respectively), whereas we found moderate agreement for Liaison (Cohen's kappa coefficient 0.58). Our study showed that Abbott and Liaison SARS-CoV-2 CLIA IgG, Roche and Siemens SARS-CoV-2 CLIA IgM/IgG/IgA assays, and LFIA Technogenetics IgG-IgM COVID-19 have good agreement in seroprevalence assessment. In addition, our findings indicate that the prevalence of IgG and total Ig antibodies against SARS-CoV-2 at the time of the study was as low as around 3%, likely explaining the amplitude of the current second wave.

## Introduction

In December 2019, an outbreak of an unexplained pneumonia was reported in the city of Wuhan, Hubei province, China. A novel coronavirus was identified as the etiological agent (named severe acute respiratory syndrome coronavirus 2—SARS-CoV-2), the associated disease defined as COVID-19 (COrona VIrus Disease, 19 stands for the year the virus was first detected).

The exponential growth of affected individuals led the WHO to declare a global pandemic; since then, the virus has greatly impacted, infecting over 80 million worldwide with more than 1.5 million deaths.

SARS-CoV-2 belongs to the coronavirus family; these are enveloped, single-stranded, positive-sense RNA viruses. Seven coronaviruses infect humans; those are classified in two genera: Alpha and Beta.

NL63 and 229E are alphacoronaviruses distantly related to SARS-CoV-2 and cause cold-like illnesses.

SARS-CoV-2 belongs to the Betacoronavirus genus, Sarbecovirus subgenus, which includes SARS-CoV responsible for the 2002/2003 outbreak and sharing 80% homology with SARS-Cov2 and MERS-CoV, responsible for the 2012 and 2015 outbreaks, respectively, and HKU1 and OC43, associated with mild upper respiratory illness, belong to other Betacoronavirus subgenera (Merbecovirus and Embecovirus, respectively) and are less related to SARS-CoV-2 (1–3).

Human coronaviruses bind different receptors. SARS-CoV-2 primarily infects pneumocytes, by binding angiotensin-converting enzyme 2 (ACE2) receptors using the transmembrane Spike (S) protein. The S protein present on the surface of the virion is one of four structural proteins (spike, nucleocapsid, membrane, and envelope) found in all coronaviruses and is responsible for both the binding to the host receptor and the fusion of the virion with the cell membrane (3). The S protein is composed by three homotrimers, each consisting of three identical polypeptide chains; each chain contains two subunits, S1 and S2. Subunit S1 makes up the majority of the S protein surface area and includes the receptor-binding domain (RBD) allowing SARS-CoV-2 to bind to the ACE2 receptor. The RBD shares only 73% similarity with SARS-CoV, and 21–25% similarity to other human coronavirus S1 subunits (4); the genetic differences in RBD dictate the viral receptor specificity.

The S2 subunit tethers the S protein to the virion membrane and includes the machinery required for virus–cell fusion (5, 6). S2 is more conserved than S1 (90% similarity with SARS-CoV-2, and 35–43% similarity with the other coronavirus S2s). Due to its location on the surface of the virus and its physiologic importance, the immunogenicity of coronavirus S protein was predicted. The serum of SARS-CoV-convalescent patients showed high titers of antibodies against the S protein (7), and in neutralization assays anti-S antibodies have shown to protect cells from SARS-CoV infection (7, 8).

Coronavirus-infected patients also exhibit antibodies with a high reactivity against the structural nucleocapsid protein (N protein) (9); this protein is very abundant, although only within the virion. Anti-N antibodies are believed to not protect cells from infection (10), since they are highly prevalent in the post-infection phase, being likely generated after digestion of viral proteins by macrophages and other antigen-presenting cells to B cells (9). Nevertheless, diagnostic assays for anti-N antibodies are easier to produce and can be useful to detect previous infection.

To address the pandemic, reliable diagnostic assays are required. Real-time reverse transcriptase-polymerase chain reaction (rRT-PCR) tests are the main diagnostic approaches and, so far, the most reliable. Real-time PCR testing requires experienced personnel and well-equipped laboratories, making mass testing of populations difficult.

To detect viral RNA, nasopharyngeal or oropharyngeal swabs are used. The limit of detection (LOD) for the molecular test can vary between 50 and 1,000 viral copies/mL (Laboratory Corporation of America Accelerated Emergency Use Authorization (EUA) Summary COVID-19 PT-PCR Test; available online at https://www.fda.gov/media/136151/download, accessed March 30, 2020). The clinical sensitivity of SARS-CoV-2 PCR tests is not well-defined, with a positive PCR test being the standard for diagnosis in most studies. Despite the high sensitivity and specificity, false-negative results at real-time PCR are an important issue. These can be due both to mutations in primers targeting regions and to the natural disease course of COVID-19. Timing of specimen collection is crucial to clinical sensitivity: early in the course of infection, both in clinical disease and in asymptomatic infections, low Ct readouts are obtained (<20), indicative of high viral loads (ranging from 10 × 10^4^ copies to >10^6^ copies/mL). Conversely, in the late phase of the infection, the viral load rapidly drops, with high Ct readouts (>32), frequently yielding non-conclusive, indeterminate results. Negative results can be obtained using assays without an adequate LOD or when little RNA is collected, making difficult the diagnosis and posing problems in contact tracing.

Finally, real-time PCR assays are not useful in identifying patients with a previously recovered SARS-CoV2 infection. Therefore, despite the high diagnostic potency, the limitations of the real-time assays make necessary the use of serological tests. The association of real-time PCR assay and serology testing improves the diagnosis of COVID-19.

Due to the drop in viral RNA, serological assays may allow to detect patients in the late stages of the infection. Serological assays could be helpful in identifying patients who have recovered from SARS-CoV2 infection, avoiding in case of contacts more expansive and time-consuming molecular tests. Serological assays can allow to deploy workers with a previous infection in high-risk settings (COVID 19 wards, ICU, etc.). Moreover, serology testing is of great importance in the seroprevalence studies, to identify donors for passive immunization or serum-transfer therapies and for the selection of the vaccine candidates.

The correlation between antibodies and the protection from reinfection is still controversial, although few cases of reinfection have been reported; serology testing of SARS-CoV2 will address this issue.

SARS-CoV-2 serological tests are already commercially available. Initially, serological tests for the detection of IgM and IgG were developed. It was believed that IgM antibodies were produced earlier than IgG; however, later studies showed that IgM and IgG antibodies are detectable with the same timing or with a short time difference (1–2 days) (11–13). More recently, assays detecting total antibodies have been developed. The detection rates of the serologic tests range from 11% in the early phase of the infection to 100% 14 days post-infection. Targets of these assays are the antibodies against the Spike protein, the S1 receptor-binding domain, and N-protein.

Due to the fast spreading of the Sars-Cov2 infection, a great number of serological assays have been developed and different methodologies have been exploited. Most are immunochromatographic assays using the lateral flow format (rapid assays), are easy to perform, do not require instruments, and use capillary blood. The relevant advantage is to obtain a diagnosis without sending samples to centralized laboratories. However, a low diagnostic performance of rapid assays has been reported, for instance in samples with a low antibody concentration, as in early phases of seroconversion, it may yield false-negative results. False-positive results, likely due to cross reactions, were frequently reported (14, 15).

Chemiluminescent tests are considered the most sensitive by methodology and provide results with great accuracy and precision. These tests are highly automated and, in some cases, allow a semiquantitative evaluation (16).

The availability of different serological assays detecting total anti-N or anti-S antibodies or the different antibody classes (IgG or IgM), the different technologies used, and poor knowledge about Sars-CoV2 infection make necessary to evaluate the diagnostic performances of the different assays commercially available, in order to improve diagnostic efficacy and seroprevalence assessment.

In the present seroprevalence study, we evaluated the performance of one lateral flow assay (Technogenetics), two chemiluminescent assays testing for total SARS-CoV-2 antibodies against N protein (Roche) or against S1 (Siemens), and two chemiluminescent assays testing IgG antibodies against N protein (Abbott) and against S protein (DiaSorin).

Moreover, we compared the seroconversion timing by analyzing the sera of confirmed SARS CoV2 patients using three different chemiluminescent immunoassays (CLIA).

## Patients and Methods

### Patients

A total of 3,185 citizens of the Campania Region were tested for anti-SARS-CoV-2 as part of a screening program. More than 90% of the tested individuals were domiciled in municipalities of the Diano Valley of the Salerno Province, with 1,168, 622, 536, 369, and 285 individuals, respectively, domiciled in Sala Consilina, Polla, Caggiano, Atena Lucana, and Auletta, respectively. The median age (interquartile range) of the entire cohort was 51 years (37–61), with 1,580 females (49.6%).

The study was conducted in accordance with the Declaration of Helsinki, and the protocol was approved by the Ethics Committee of the University of Naples “Federico II” (Project Identification Code 140/20/ESCOVID19). The patients/participants provided their written informed consent to participate in this study.

Serum samples were collected, refrigerated, and transported to the laboratory for testing. All samples were tested using the different analyzers.

### Methods

The Elecsys Anti-SARS-CoV-2 is an electrochemiluminescence immunoassay (ECLIA) detecting total antibodies including IgG using a recombinant protein representing the nucleocapsid antigen (N antigen). Results are reported as a cutoff index (COI) and interpreted as negative (COI < 1.0) or positive (COI ≥ 1.0). Positive and negative controls were prepared using pooled patient samples according to manufacturer instructions. Controls and patient samples were analyzed on a Cobas e411 instrument (Roche) according to manufacturer instructions.

The ADVIA Centaur COV2T assay is a one-step antigen sandwich immunoassay using acridinium ester chemiluminescent technology, in which antigens are bridged by antibodies present in the patient sample. The solid phase contains a preformed complex of streptavidin-coated microparticles and biotinylated SARS-CoV-2 recombinant S antigens. Results are determined according to the Index Value. Samples were considered reactive: ≥1.0 Index or non-reactive: <1.0 Index. Samples were analyzed using the ADVIA Centaur XPT instrument.

The Abbott SARS-CoV-2 IgG test assay uses nucleocapsid protein for antibody detection. The assays were performed on an Abbott Architect i1000 analyzer following the manufacture instructions. Samples with a signal-to-cutoff (S/CO) ratio ≥1.4 were considered positive.

LIAISON SARS-CoV-2 S1/S2 IgG (DiaSorin) is an indirect chemiluminescent immunoassay for the quantitative detection of IgG antibodies against S1/S2 proteins [cutoff of 12 arbitrary units (AU)/mL, classifying gray zone results of 12–15 AU/mL as positive].

The subjects were also analyzed with COVID-19 IgM/IgG Rapid Test Technogenetics, an immunochromatographic test for the qualitative determination of IgM and IgG class antibodies against COVID-19 in human serum, plasma, and whole blood. A specificity of 99.4% and a sensitivity of 100% at day 16 after infection is reported by the manufacturer.

### Statistical Analysis

Descriptive data of COVID-19 positivity were expressed as absolute number and prevalence. The prevalence measured by each of the serological tests was estimated by computing the ratio between the positive cases and the total number of tested subjects belonging to each considered category. Agreement among the Liaison Igg, Abbott IGG, Technogenetics IGG, Siemens, and Roche tests was measured as overall raw agreement and Fleiss' kappa coefficient. Agreement between the COVID-19 rapid IGG test and each IGG serological test (Liaison, Abbott, and Technogenetics) and between the rapid test and the Roche and Siemens tests was measured as absolute count, percentage of overall raw agreement (on positive and negative cases), and Cohen's kappa coefficient. Fleiss' kappa and Cohen's kappa coefficients can be interpreted as follows: <0.20, poor agreement; 0.21–0.40, fair agreement; 0.41–0.60, moderate agreement; 0.61–0.80, good agreement; and >0.81, very good agreement (17). All statistical analyses were performed using the R statistical environment, version 4.0.2 (18).

## Results

**Figure 1:**
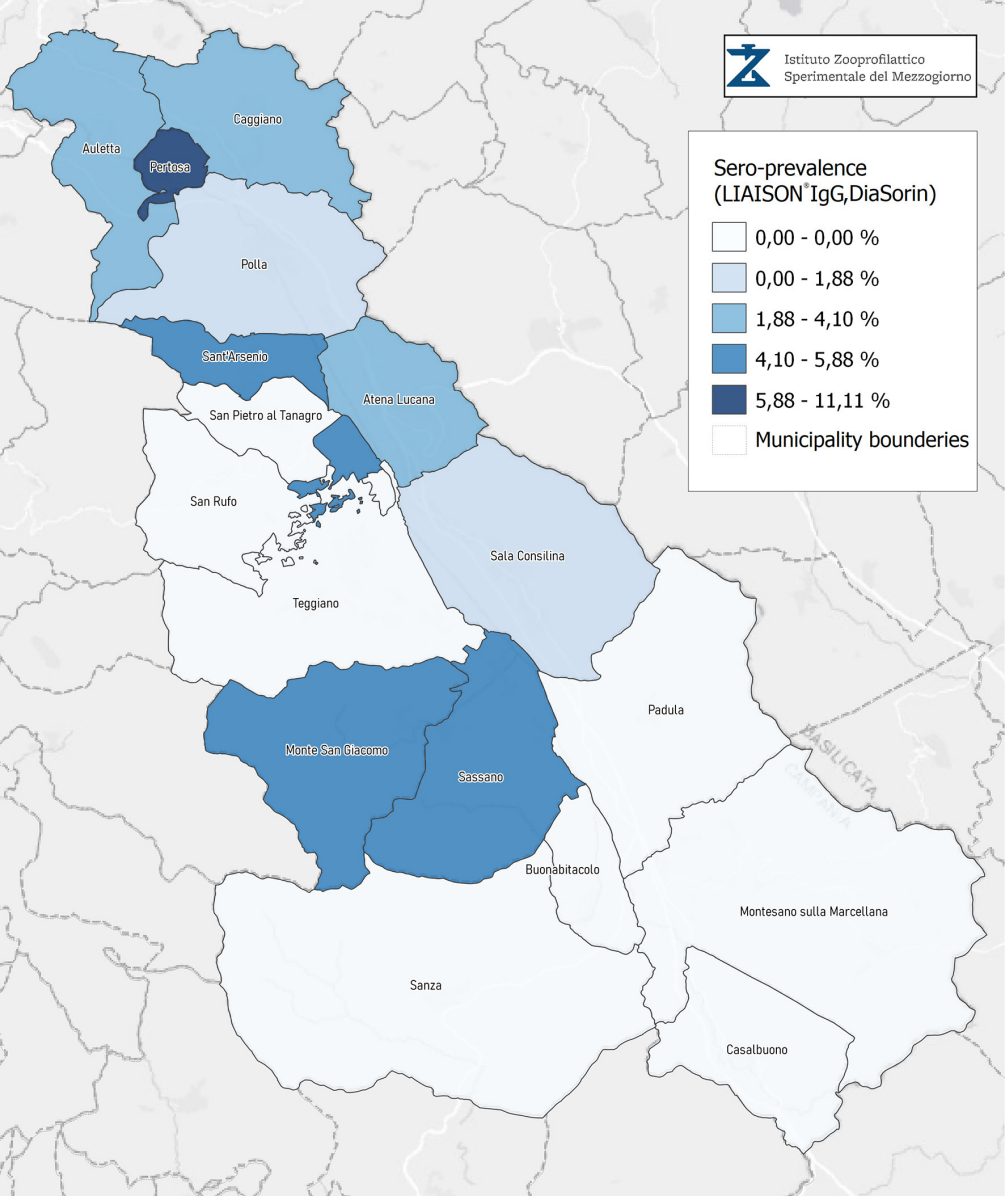
Seroprevalence assessed in Diano Valley municipalities by Liaison IgG Diasorin.

A total of 3,185 citizens dwelling in multiple municipalities of the Campania Region were tested for anti-SARS-CoV-2, as part of an institutional screening program promoted (IZSM). More than 90% of the tested individuals were domiciled in municipalities of the Diano Valley of the Salerno Province; the geographical distribution is shown in Figure 1 and Table 1. The median age (interquartile range) of the entire cohort was 51 years (37–61), with 1,580 females (49.6%).

**Table 1:** Absolute number and prevalence of COVID-19 infections as measured by the different serological tests grouped by age, sex, and area.

**Demographic information (*N* = 3,083)**	**Positive cases (prevalence)**					**Agreement**	
	**Liaison IgG**	**Abbott IgG**	**Technogenetics IgG**	**Siemens total Ig**	**Roche total Ig**	**Agreement (%)**	**Fleiss' kappa**
**Age**							
<18	5 (3.60)	4 (3.15)	3 (2.26)	5 (3.60)	5 (3.60)	98.53	0.80
18–65	60 (2.48)	52 (2.18)	49 (2.09)	44 (1.82)	43 (1.78)	99.16	0.61
>65	12 (1.94)	11 (1.82)	14 (2.32)	9 (1.45)	11 (1.82)	98.89	0.71
**Sex**							
Female	39 (2.47)	35 (2.27)	34 (2.23)	33 (2.09)	30 (1.90)	98.50	0.67
Male	38 (2.38)	32 (2.03)	32 (2.03)	25 (1.56)	29 (1.81)	98.45	0.60
**Area**							
Atena Lucana	12 (3.27)	11 (3.15)	7 (1.91)	7 (1.91)	9 (2.45)	98.39	0.65
Auletta	7 (2.46)	4 (1.44)	7 (2.46)	4 (1.44)	5 (1.76)	98.88	0.79
Caggiano	22 (4.13)	20 (3.79)	9 (1.78)	18 (3.38)	17 (3.19)	97.58	0.64
Polla	10 (1.61)	6 (0.98)	6 (0.98)	4 (0.64)	3 (0.48)	98.79	0.37
Sala Consilina	22 (1.88)	23 (2.00)	33 (2.90)	25 (2.14)	25 (2.14)	98.71	0.70
Total	76 (2.48)	71 (2.34)	66 (2.14)	58 (1.89)	58 (1.89)	98.42	0.64

*In addition, raw overall agreement and Fleiss kappa agreement indices are reported*.

**Figure 2:**
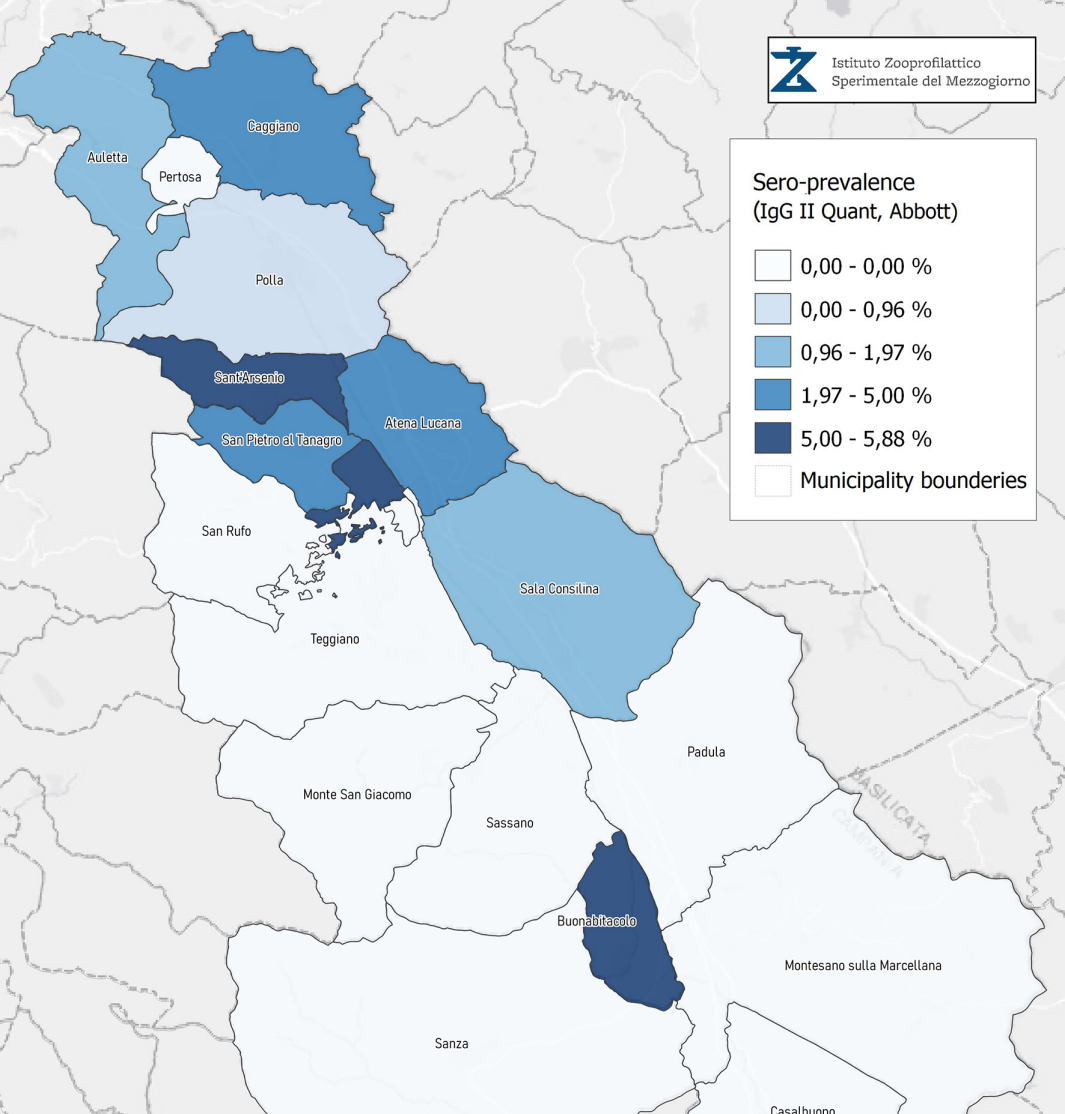
Seroprevalence assessed in Diano Valley municipalities by IgG II Quant Abbott.

**Figure 3:**
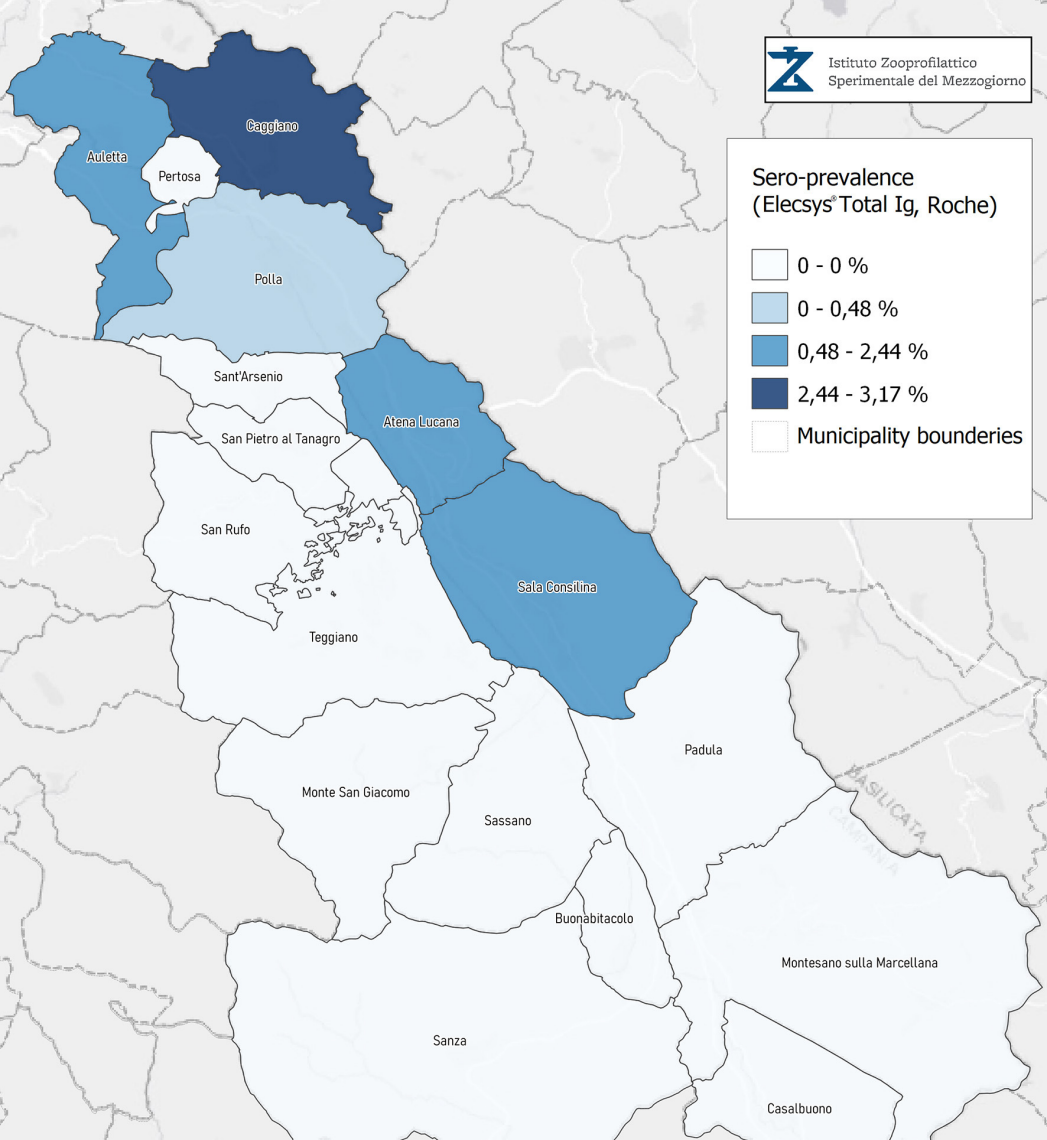
Seroprevalence assessed in Diano Valley municipalities by Elecsys Total Ig Roche.

**Figure 4:**
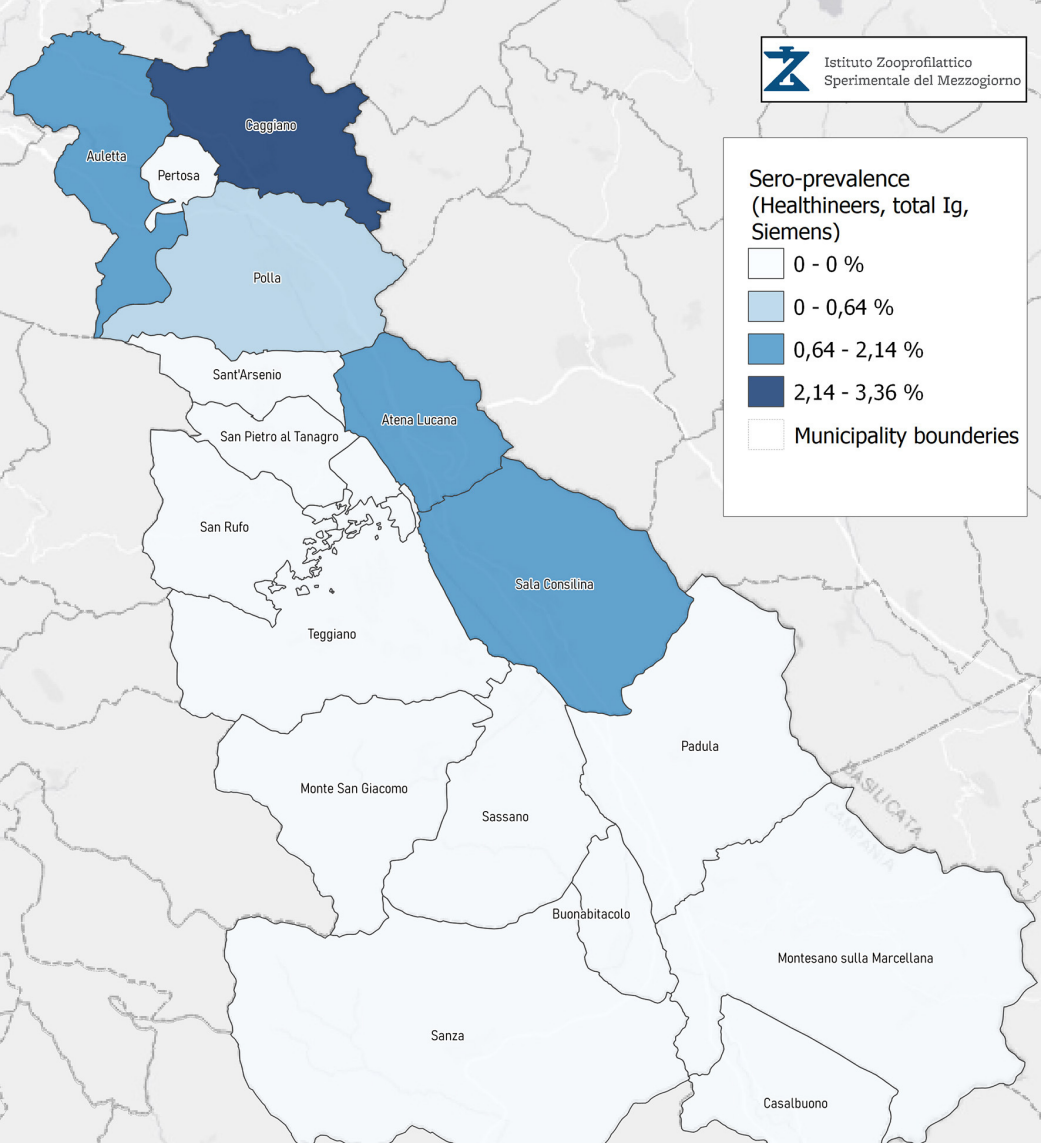
Seroprevalence assessed in Diano Valley municipalities by Healthineers, total Ig Siemens.

Seroprevalence in the entire cohort was 2.41, 2.10, 1.82, and 1.85% according to the Liaison IgG (Figure 1), Abbott IgG (Figure 2), Roche (Figure 3), and Siemens (Figure 4) total Antibodies tests (see Table 1).

Seroprevalence appeared slightly higher when assessed using Liaison and Abbott IgG tests as compared to Siemens and Roche tests. Seroprevalence appeared to be higher in younger citizens, independently on the test used. Finally, the highest seroprevalence was found in municipalities of Caggiano and Atena Lucana, independently on the test used. A total of 3,185 citizens were tested for SARS-CoV-2 antibodies using anti-IgG and anti-IgM rapid tests on capillary blood. A total of 63 (2%) and 14 (0.4%) individuals tested positive on anti-IgG and IgM rapid antibody detection tests. When we explored the agreement among the rapid tests and the serologic assays (Tables 2, 3), we reported good agreement for Abbott, Siemens, and Roche (Cohen's kappa coefficient 0.69, 0.67, and 0.67, respectively), whereas we found a moderate agreement for Liaison (Cohen's kappa coefficient 0.58).

**Table 2:** Comparison of rapid IgG assay and anti-S IgG Abbott and DiaSorin assays.

	**Rapid IgG–**	**Rapid IgG+**	**Agreement (%)**	**Cohen's kappa**
Liaison–	3,055	21	97.45	0.58
Liaison+	36	41		
Abbott–	3,031	17	98.84	0.69
Abbott+	22	45		
Technogenetics–	2,978	33	97.47	0.42
Technogenetics+	39	27		

*Agreement between the rapid test and the Liaison, Abbott, and Technogenetics IgG tests. The results are expressed as absolute count, raw overall agreement, and Cohen's kappa coefficient*.

**Table 3:** Agreement between the rapid test and the Roche and Siemens tests.

	**Rapid–**	**Rapid +**	**Agreement (%)**	**Cohen's kappa**
Roche–	3,081	26	98.33	0.67
Roche+	16	43		
Siemens–	3,082	26	98.36	0.67
Siemens+	15	43		

*The results are expressed as absolute count, raw overall agreement, and Cohen's kappa coefficient*.

## Discussion

Increased mass testing and contact tracing together with physical distancing and restriction of movement were efficacious in decreasing transmission rates of SARS-CoV-2 (19). Unfortunately, this kind of measures has unfavorable societal and economic impacts potentially resulting in significant recession; therefore, alternative strategies to control the pandemic are required.

An approach to maintaining epidemiological vigilance and allowing a fast response to the rise of viral infections is to identify and quantitate people with immunity against SARS-CoV-2 in the whole population. This approach could discriminate immune people as health-care workers allowing to reopen activities and borders and follow the development of the herd immunity. Different methods for serological tests are currently available (20).

To strengthen surveillance systems, it is therefore important to evaluate serological assays that can be used in large-scale studies. In this sero-epidemiological study for SARS-CoV-2, we evaluated different serological tests in a large study population of Diano Valley (Campania Region).

Our findings indicate that the prevalence of IgG and total Ig antibodies against SARS-CoV-2 at the time of the study was as low as around 3%, likely explaining the amplitude of the current second wave.

Since the study was designed to obtain data on the Diano Valley area, we were able to reveal differences among the different urban settlements. Caggiano and Atena Lucana showed the highest prevalence around 4%, whereas in Polla the lowest prevalence was observed, confirming a difference in the spread of viral infection among these settlements. Further studies are required to explain these differences. A limitation of our study is the lack of a comparison with epidemiological data in emergency time. However, to our knowledge, this study is one of the largest population-based SARS-CoV-2 seroprevalence studies in Southern Italy with more than 3,000 participants.

The use of two IgG antibody tests, two total Ig antibody tests directed against N or S antigens, and a rapid test allows us to specify a range of seroprevalence between 0.48 and 4.13%.

These estimates clearly indicate a lower magnitude of seroprevalence in Southern compared to Northern Italy (21, 22), partially explaining the extent of the second wave in the Campania region.

As reported for other coronaviruses (23, 24), prevalence was higher in younger citizens both when using the point-of-care test and when using the CLIA. The lower prevalence in young people might be explained on the base of a more efficient immunological response (25, 26). A lower nasal gene expression of the angiotensin-converting enzyme 2 receptor in younger might also explain this lower seroprevalence (27).

Our results also highlighted the performances of different commercial assays to assess the rate of infection in a target population. At variance with other studies, we observed a good agreement for Abbott, Siemens, and Roche automated immunometric assays and Technogenetics rapid commercial assays. However, previous studies compared rapid assays and CLIAs in symptomatic SARS-Cov-2 patients. In these patients, anti-SARS-Cov-2 antibodies are observed 5 days or more from the appearance of the symptoms. In our screening study, we assessed the seroprevalence of a population without any section, thereby detecting previous and resolved SARS-Cov-2 infections. Our data demonstrate that the Technogenetics rapid assays can be useful for epidemiological studies, whereas the assessment of the diagnostic performance of this assay requires further studies.

Interestingly, the seroprevalence appeared slightly higher using IgG anti-S (Liaison assay) and anti-N (Abbott assay) with respect to anti-total Antibodies Siemens (anti-S) and Roche (anti-N). This effect is likely due to a less efficient detection of IgG in total assays compared to IgG-specific assays, as previously reported. A recently published meta-analysis demonstrated that IgG tests had better sensitivity when the samples were taken a week after the onset of symptoms (28). Accordingly, IgM antibodies showed lower specificity than IgG (28).

Several factors could affect the ability of antibody tests to identify infected people, including quality of the sample, low antibody levels, and timing of the test (29). Kinetic studies (30, 31) showed that IgM reaches a peak between days 5 and 12 and then drops, whereas IgG reaches a peak after day 20 or so as IgM antibodies disappear.

Evidence indicates that total antibody tests seem to be more sensitive than single-antibody testing (28). Furthermore, S-based tests were reported as more specific due to poor cross-reactivity with low conserved regions of spike proteins of other coronaviruses (SARS-CoV) (32). In addition, it has been demonstrated that tests detecting antibodies anti-S antigen are more sensitive with respect to test detecting anti-N antibodies, probably since the immune response against S antigen seems earlier with respect to the response to N antigen (28).

The sensitivity and specificity of antibody tests are relevant issues for both diagnosis and epidemiological surveillance. False-positive results may allow to consider immune people who have never been infected and may alter prevalence estimates, mortality rate, and herd immunity assessment.

False-negative findings may prevent to contain viral spread.

Meta-regression analysis showed that CLIAs showed comparable sensitivity (~90%) but slightly decreased specificity (95–98%) with respect to ELISA tests (higher than 99% and sensitivity ~93%). The lateral flow immunoassay test showed specificity as high as that of the ELISA test (~99%) and a lower sensitivity (~80%).

Accordingly, our results suggested that despite the suboptimal sensitivity, antibody tests could integrate nucleic acid testing both in the diagnosis of SARS-Cov-2 infection and in the assessment of seroprevalence in the entire population (13). When designing seroprevalence studies, attention should be paid to the sensitivity and specificity of the antibody's tests. In the diagnostic assessment, a combined strategy as retesting a negative result with a different method to ameliorate specificity could be advantageous.

In addition, some practical aspects should be considered: for wide screening, completely automated CLIA methods could be advantageous, although rapid tests as immunochromatographic cards should be useful (when centralized laboratories are not available).

Further studies on a large population are needed to compare serological tests and nucleic-acid testing to better define which is the best approach for diagnosis and which for seroprevalence assessment.

## Data Availability Statement

The raw data supporting the conclusions of this article will be made available by the authors, without undue reservation.

## Ethics Statement

The study was conducted in accordance with the Declaration of Helsinki and the protocol was approved by the Ethics Committee of the University of Naples Federico II (Project Identification Code 140/20/ESCOVID19). The patients/participants provided their written informed consent to participate in this study.

## Author Contributions

PC, DT, and GPo: conception and design, analysis, interpretation of data, critical revision of the manuscript for important intellectual content, and supervision. AG, BP, CB, DD, MCC, LV, GL, AnC, AP, GB, VF, LA, MT, MC, PR, TS, AF, OC, AlC, GPe, and MD'A: acquisition of data. DT and GPo: drafting of the manuscript. DP: statistical analysis. PC and GPo: administrative, technical, or material support. All authors contributed to the article and approved the submitted version.

## Conflict of Interest

The authors declare that the research was conducted in the absence of any commercial or financial relationships that could be construed as a potential conflict of interest.
